# 3D printing-based Ganz approach for treatment of femoral head fractures: a prospective analysis

**DOI:** 10.1186/s13018-019-1383-7

**Published:** 2019-10-29

**Authors:** Jinwu Wang, Leyi Cai, Linzhen Xie, Hua Chen, Xiaoshan Guo, Kehe Yu

**Affiliations:** Department of Orthopaedics Surgery, The Second Affiliated Hospital and Yuying Children’s Hospital of Wenzhou Medical University, NO.109, XueYuan West Road, Luheng District, Wenzhou, 325000 Zhejiang Province People’s Republic of China

**Keywords:** 3D printing, Ganz approach, Femoral head fractures, Avascular necrosis, Heterotopic ossification

## Abstract

**Background:**

Femoral head fractures are uncommon injuries. Open reduction and internal fixation (ORIF) of femoral head fracture is the preferred treatment for most patients. There are several surgical approaches and treatments for this difficult fracture. However, the optimal surgical approach for the treatment of femoral head fracture remains controversial. Meanwhile, the operation is difficult and the complications are numerous. We prospectively reviewed patients with femoral head fractures managed surgically through the 3D printing-based Ganz approach to define a better approach with the least morbidity.

**Patients and methods:**

Between 2012 and 2017, a total of 17 patients were included in this study. An exact 1:1 3D printing model of the injured hip side was fabricated for each patient and simulated surgery was finished preoperative. The surgical approach was performed as described by Ganz. Functional assessment was performed using the modified Merle d’Aubigne scores. The reduction of the fracture was evaluated according to Matta’s criteria. The incidence of complications, such as heterotopic ossification (HO) and avascular necrosis (AVN), and the need for additional surgery were also documented.

**Results:**

Twelve of 17 patients (four females and eight males) were available for 2 years follow-up. The mean follow-up was 35 months (25–48 months). Average age for the 12 patients was 39.9 ± 12.2 years. According to the Pipkin classification, four patients were type I fracture, three patients were type II fracture, and five patients were type IV fracture. The mean operative time was 124.2 ± 22.1 min, and the estimated blood loss was 437.5 ± 113.1 ml. According to Merle d’ Aubigne scores, excellent results were achieved in six of the 12 patients; four good and two poor results occurred in the rest of the patients. On the radiograph evaluation, fracture reduction was defined as anatomical in eight patients, and imperfect in four. Most patients had good outcomes and satisfactory hip function at last follow-up. Almost all great trochanteric osteectomy healed uneventfully. One patient developed symptomatic AVN of the femoral head and underwent THA at 3 years. After THA, she regained a good hip function with the ability to return to work and almost no reduction in sports activities. Heterotopic ossification was found in four cases (type I-1, type II-2, and type III-1).

**Conclusions:**

The 3D printing-based Ganz approach provides a safe and reliable approach and satisfactory results of treatment in femoral head fractures. Using 3D printed model for the fracture of the femoral head, the fracture can be viewed in every direction to provide an accurate description of fracture characteristics, which contributes to make a reasonable surgical plan for patients. In addition, the 3D printing-based Ganz approach can obtain excellent surgical exposure and protection of the femoral head blood supply, reduce the operation time and intraoperative blood loss, make the precise osteotomy, anatomically fix the intra-articular fragments, and effectively reduce postoperative complications.

**Trial registration:**

We register our research at http://www.researchregistry.com. The Unique Identifying Number (UIN) from the Research Registry of the study is researchregistry4847.

## Introduction

**Fig. 1:**
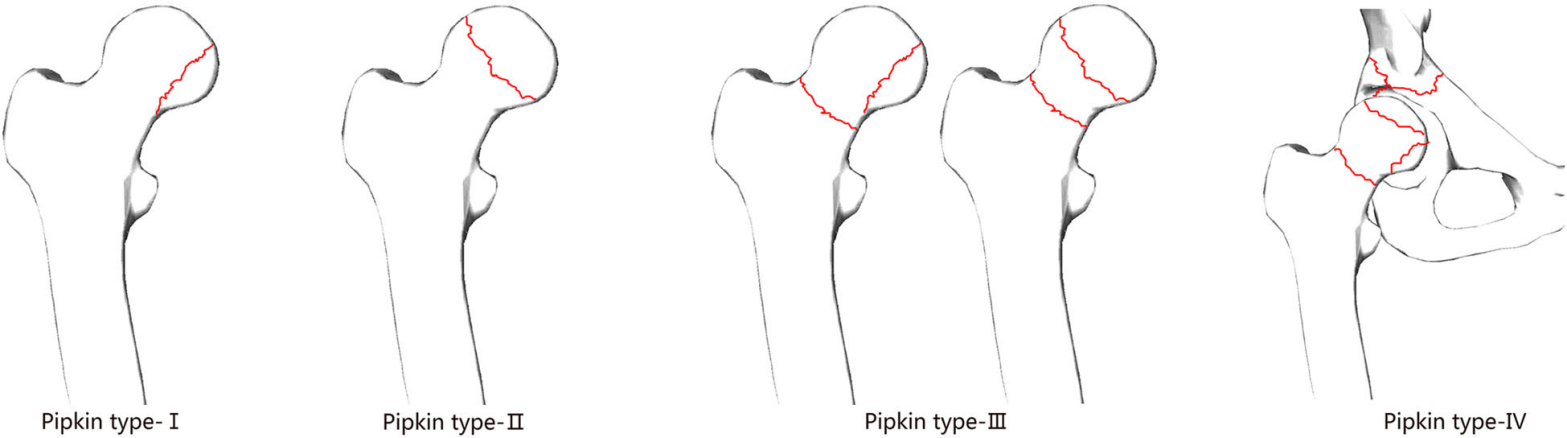
Pipkin classification of femoral head fractures with posterior hip dislocations. Types I and II are distinguished by the position of the fracture in relation to the fovea. Type I is below the fovea with the fracture outside of the weight-bearing joint-parts, whereas type II fractures involve the more cranial, weight-bearing parts. Type III is any fracture of the head in combination with a femoral neck fracture. Additional fractures of the acetabulum are classified as type IV

Fracture of the femoral head was described by Birkett [1] in 1869 firstly, who found this fracture during dissection of a cadaver. Due to the intrinsic anatomical stability of the hip, most of these injuries were caused by high-energy trauma, typically including falls from a significant height or car accidents (such as pedestrians being run over and collisions) [2]. Park et al. [3] found traffic accidents accounted for 93.9% of femoral head fractures, Kelly and Yarbrough [4] suggested an incidence of 92.6% and Pipkin [5] reported traffic accidents as the cause of femoral head fractures in about 92% of all such fractures. Approximately two thirds of patients were young adults, occurring in as many as 75% of the cases [6]. About 5~15% of posterior hip dislocations have been reported to be associated with femoral head fractures [7]. The most widely used classification was that of Pipkin [5] which was based on the location of the femoral head fracture in relation to the fovea and additional lesion on the femoral neck or acetabulum as shown in Fig. 1. The absence of randomized controlled data and a validated outcome instrument have contributed to the lake of absolute recommendations and indications for the most appropriate treatment of these injuries. There is still no consensus on the management of injuries: whether to treat these fractures operatively or non-operatively, whether to fix or excise the head fragment, or which surgical approach to use.

The fracture itself as well as posttraumatic changes such as heterotopic ossification (HO), avascular necrosis (AVN) of the femoral head, and secondary osteoarthritis might lead to a restriction in hip function and permanent disability even in young patients [8]. The principles of treatment included prompt reduction of the associated hip dislocation, early anatomic reduction, rigid fixation of large fragments, restoration of hip congruency, and stability and removal of small and comminuted intra-articular fragments [9].

**Fig. 2:**
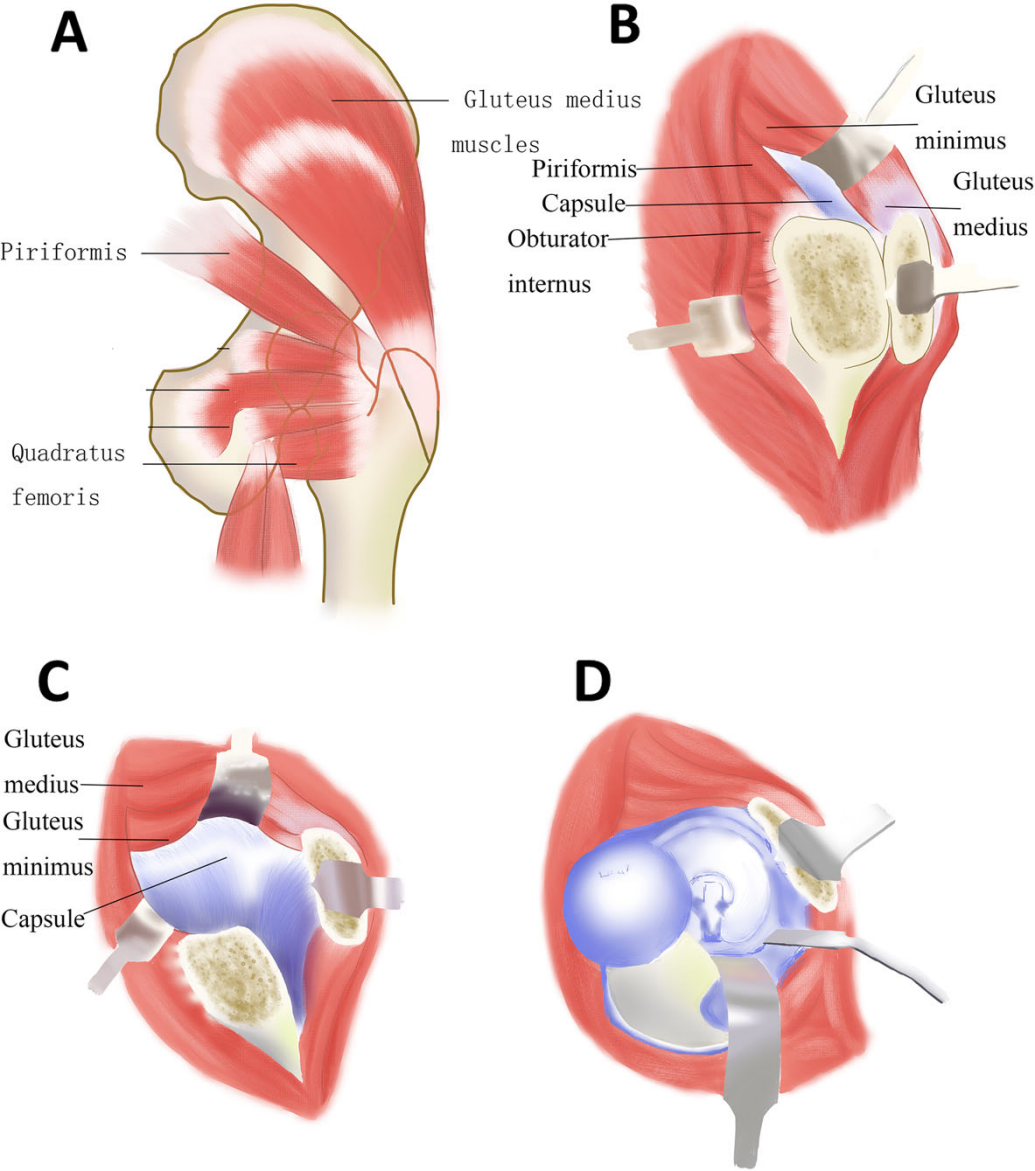
Osteosynthesis of a femoral head fracture using the Ganz approach. **a** The position of the trochanteric osteotomy: The osteotomy exits just anterior to the most posterior insertion of gluteus medius proximally and the entire origin of vastus lateralis remains on the trochanteric fragment distally. **b** After the osteotomy of the trochanter, the osteotomized trochanter fragment, including the tendon of gluteus minimus, is reflected anteriorly. Through blunt separation, the tissue space between the tendon of piriformis and gluteus minimus is enlarged and gluteus minimus contracts upward, which contributes to adequately expose the capsule. **c** Make a Z-shaped capsule incision to further rotate outward and flex the femur. **d** After dislocation of the femoral head, lower the knee and rotate the affected leg, which can make the fracture can easily visualized, reduced, and stabilized

The matter of which operative approach should be used for the surgical treatment of femoral head fractures remains controversial [10]. The anterior, posterior, and lateral approaches have been reported, with advantages and disadvantages. Previous studies [11, 12] strongly objected to choose an anterior approach and advocated the use of posterior Kocher–Langenbeck approach, deeming that the former would damage any residual blood supply of the femoral head. However, some anatomical researches [13–15] revealed that the medial femoral circumflex artery was the main source of femoral head vascular supply and specifically its deep branch, while the lateral femoral circumflex artery contributed a little to it. Subsequently, studies [16–19] carried out the anterior Smith-Petersen approach with relatively satisfactory results, emphasizing that it offered easier access to and fixation of the fractured head. Recently, a posterior-based approach with a trochanteric flip osteotomy and a surgical hip dislocation have been advocated for the management of the femoral head fractures which is first reported by Ganz et al. in 2001 with 213 hips [20]. This approach preserves the deep branch of the medial femoral circumflex artery (MFCA) and at the same time allows unimpaired complete visibility of the femoral head, permitting reduction and fixation of the fragments under direct visual control as shown in Fig. 2.

In recent years, along with the development of digital medicine in clinical treatment, 3D printing technology has achieved a quantum leap from virtual simulation to the real-world clinical application, which overcomes the two-dimensional limitation of CT and MRI data and presents a real anatomical structure [21, 22]. Moreover, this technology has been widely used in various fields, including dentistry, anatomical models, medical devices, tissue engineering and regenerative medicine, engineered tissue models, and drug formulation [23]. Currently, various kinds of biological materials have been developed continuously, which were expected to be used for 3D printing [24–26]. And a variety of 3D printing models and their fabrication processes have been applied, which were prepared in a form of powders, granules, dense or porous scaffolds, and bioactive coatings on metal prosthesis [27–29]. Our previous research has proven the advantages of 3D printing technology in orthopedic surgeries [30–32], such as shortening the operation time and reducing the intraoperative blood loss. Tack et al. [33] also found that 3D-printing technology reduced operation time in 46% of the studies, 72% mentioned improved medical outcomes, and 76% of the studies mentioned that the printed part had good accuracy. What is more, it also can assist in accurate preoperative planning, as well as surgical strategy simulation, and enhance communication with patients.

We have been using the Ganz approach assisted by 3D printing technology to operate incongruent hips with displaced femoral head fractures at our institution from 2012. In this prospective trial, we report our results with the approach in 12 Pipkin fractures with a minimum follow-up of 2 years.

## Patients and methods

We performed a prospective analysis of all patients with a femoral head fracture who were treated operatively with open reduction and screw fixation by use of the 3D printing-based Ganz approach at our institution between 2012 and 2017. The work had been reported in line with the STROCSS criteria [34]. There were a total of 21 femoral head fractures during the study period. We surgically treated 17 patients who were younger than 60 years old under this approach, because three patients were older than 60 years old and had been treated with total hip arthroplasty (THA). Conservative treatment was only justifiable for one patient because the post-reduction CT demonstrated an anatomic reduction of the head fragments [35]. Each fracture was classified according to the Pipkin classification.

All patients presented to our emergency department, and after initial evaluation according to the Adult Trauma Life Support (ATLS™) guidelines [36], immediate closed reduction (< 6 h for each patient) of the hip fracture dislocation was attempted under general anesthesia. There was a patient with irreducible dislocation with the femoral head buttonholed through the capsule and impaled against the posterior rim of the acetabulum. This patient indicated open reduction and internal fixation with emergency surgery; thus, the patient was excluded due to the lack of time for 3D printing preoperative. All the rest patients received a CT scan to evaluate the quality of reduction. Operative treatment was indicated for fractures displaced 2 mm or more in CT scan. Two patients were lost to follow-up, and two patients were excluded because of follow-up of less than 2 years, leaving a total of 12 patients as the study population. Patient age at surgery, sex, side of injury, operative time, and postoperative complications were recorded in a database. Patients were asked to give their informed consent to the use of an unconventional approach when eligible according to the selection criteria. This study was approved by the Institutional Review Board of our hospital.

### Preparation of the 3D model and surgical simulation

We received computed tomography (CT) scans of the die-punch fractures from the Star PACS system (INFINITT, Seoul, South Korea) of our institution. The original CT data were stored in DICOM format and 3-dimensional (3D) reconstructed using Mimics software v20.0 (Materialise, Leuven, Belgium), positioning by adjusting the threshold to reveal the intact structures of femur and the bones around the hip joint. The 3D model of the injured hip was produced using Unite Boolean calculation and further processing for the noise reduction and smoothing of the hip joint. The design data was then imported into the 3D printing software (Cura Software v15.02) in STL format. After a 3D digital model was formed, we saved it in Coed format and exported it to a 3D printer (3D ORTHO Waston Med, Inc., Changzhou, Jiangsu, China). Finally, the exact 1:1 model of the injured hip side was fabricated. The main components of the 3D printing model (Anhui Yinke Technology Co., Ltd.) were 30~70% epoxy resins, 15~50% acrylates, and 2~10% photoinitiator. And its mechanical properties were as follows: Hardness was 76~88 shore D, flexural strength was 69~74 Mpa, heat deflection temperature was 39~52 °C, and the density was 1.12~1.18 g/cm^3^.

The structural characteristics of the fracture were clear from the 3D printed model. Firstly, a classic trochanteric osteotomy was accomplished; the thickness of the osteotomized fragment was 1.5 cm [37]. Then, we mimicked the intraoperative reduction and fixation maneuver accurately on the models. Moreover, suitable plates and screws were chosen in the real-size hip model. The ideal length, location, and orientation were placed on the model. Then, the X-ray of the model would be taken for checking the proper position of the plate and the screws, which would be sterilized and stored for later use in surgery. In addition, the 3D printed model could be used intraoperatively as a reference for anatomical reduction of the fracture.

### Surgical technique

The operation was started under general anesthesia or spinal anesthesia. Surgery was performed with the patients in the lateral decubitus position on the contralateral side using a standard operation table. Sterilization and drape of the incision centered on the femoral trochanter. In addition, a sterile bag was fixed on the ventral side of the patient for positioning the leg during hip dislocation. The surgical approach was performed as described by Ganz et al. [20]. An incision was made from the posterosuperior edge of the greater trochanter extending distally to the posterior border of the ridge of vastus lateralis. The trochanteric flip osteotomy (V-shaped myofascial flap) was carefully performed staying laterally to the insertion of the short external rotators. The thickness of the osteotomised fragment should not exceed 1.5 cm to protect the MFCA which had been simulated on a 3D printed model.

The trochanteric flip was slided anteriorly after releasing the origin of the gluteus minimus. A Z-shaped anterosuperior capsulotomy was performed when the capsule was intact, or completed if there was a partial capsular rupture. This interval was safe regarding the risk of damage to the deep branch of the medial circumflex artery [13]. Then, the femoral head could be dislocated after the leg was brought into further flexion and external rotation. The leg was then placed into the sterile bag placed on the opposite side of the operating table. The femoral head and the acetabulum were inspected with a full 360° view by manipulating the leg because of the gap of up to 11 cm between them. The next step was debridement and removal of small osteochondral fragments and free cartilage flaps. In cases where larger head fragments did still have soft tissue attachment, this was mostly via the inferior retinaculum. Those attachments should be preserved in any circumstances because this connection could provide valuable blood supply to the fragment. For estimation of blood supply to the femoral head, we speculated that bleeding of the surfaces of the cancellous bone after trimming osteophytes on the periphery of the head were further signs of satisfactory vascularity.

**Fig. 3:**
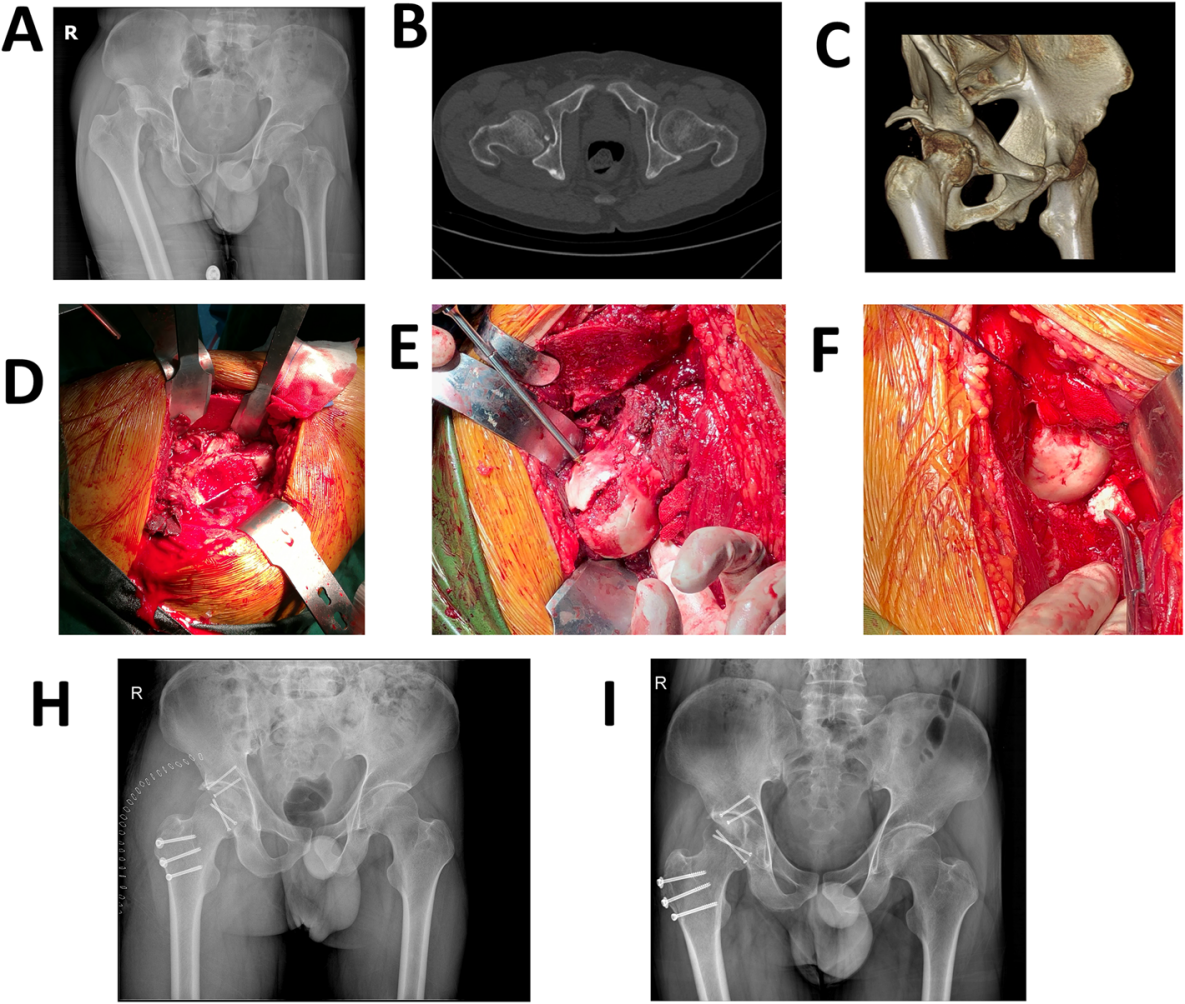
This was a 58 years old male with type IV fracture caused by car accident. **a** The femoral head fracture, posterior dislocation of the hip, and posterior acetabular wall fracture. **b**, **c** The CT scan after reduction which showed detail of the fracture. **d** The osteotomy of the trochanter: the osteotomized trochanter fragment is reflected anteriorly. **e**, **f** The reduction and fixation with femoral head fracture and acetabular fracture. **h** The X-ray after operation. **i** The X-ray of 1 year after operation, which showed the fracture has healed and there is no AVN

**Fig. 4:**
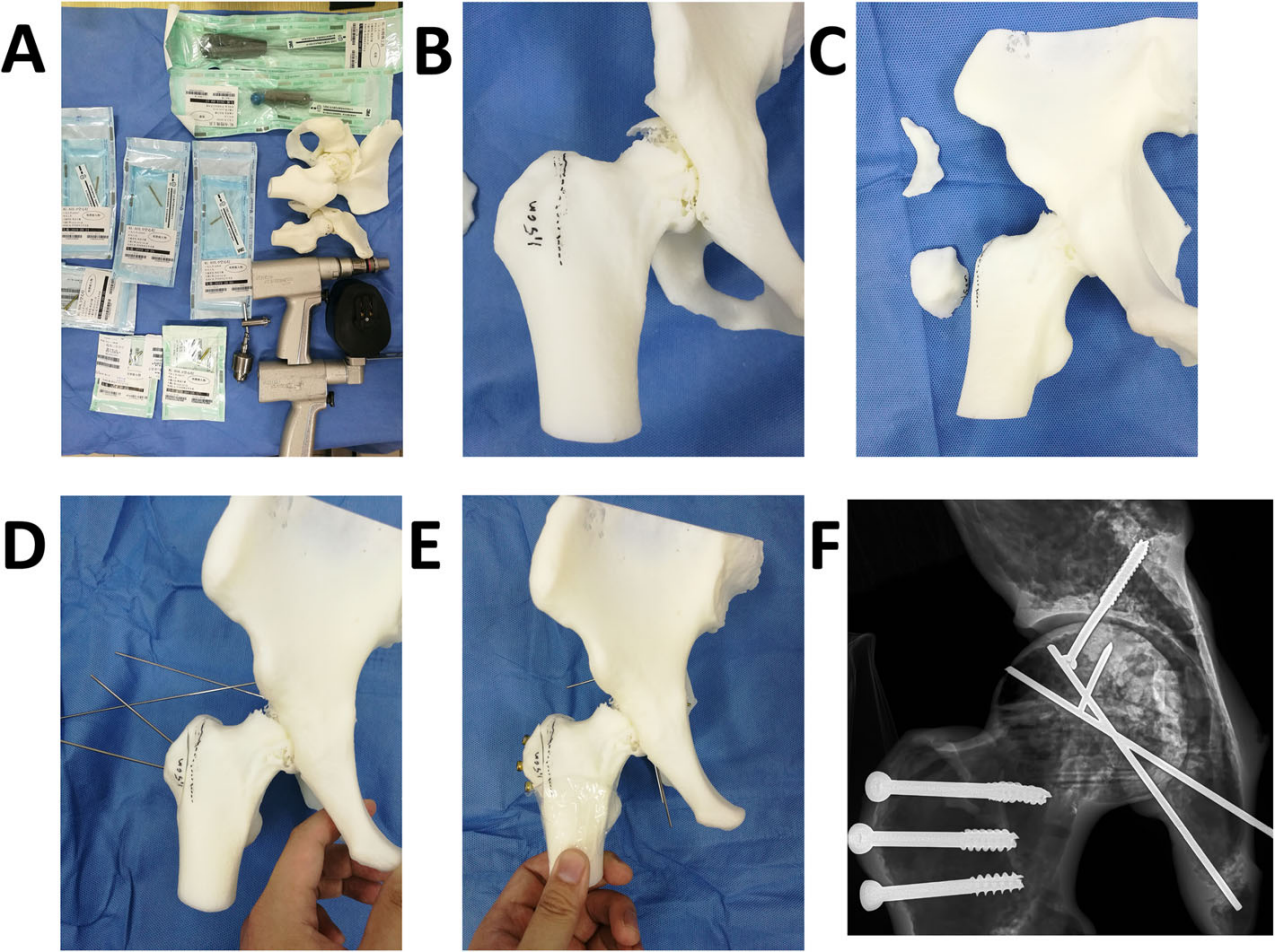
Simulation of the surgery in 3D printing model for the patient of Fig. 3. **a** Preparation of simulative surgery. **b** Mark at the osteotomy line. **c** Complete the femoral trochanter osteotomy and separate the fracture fragment of the posterior wall of the acetabulum. **d**, **e** Reduction and fixation with suitable length screws and Kirschner wire. **f** X-ray after the simulative surgery

**Fig. 5:**
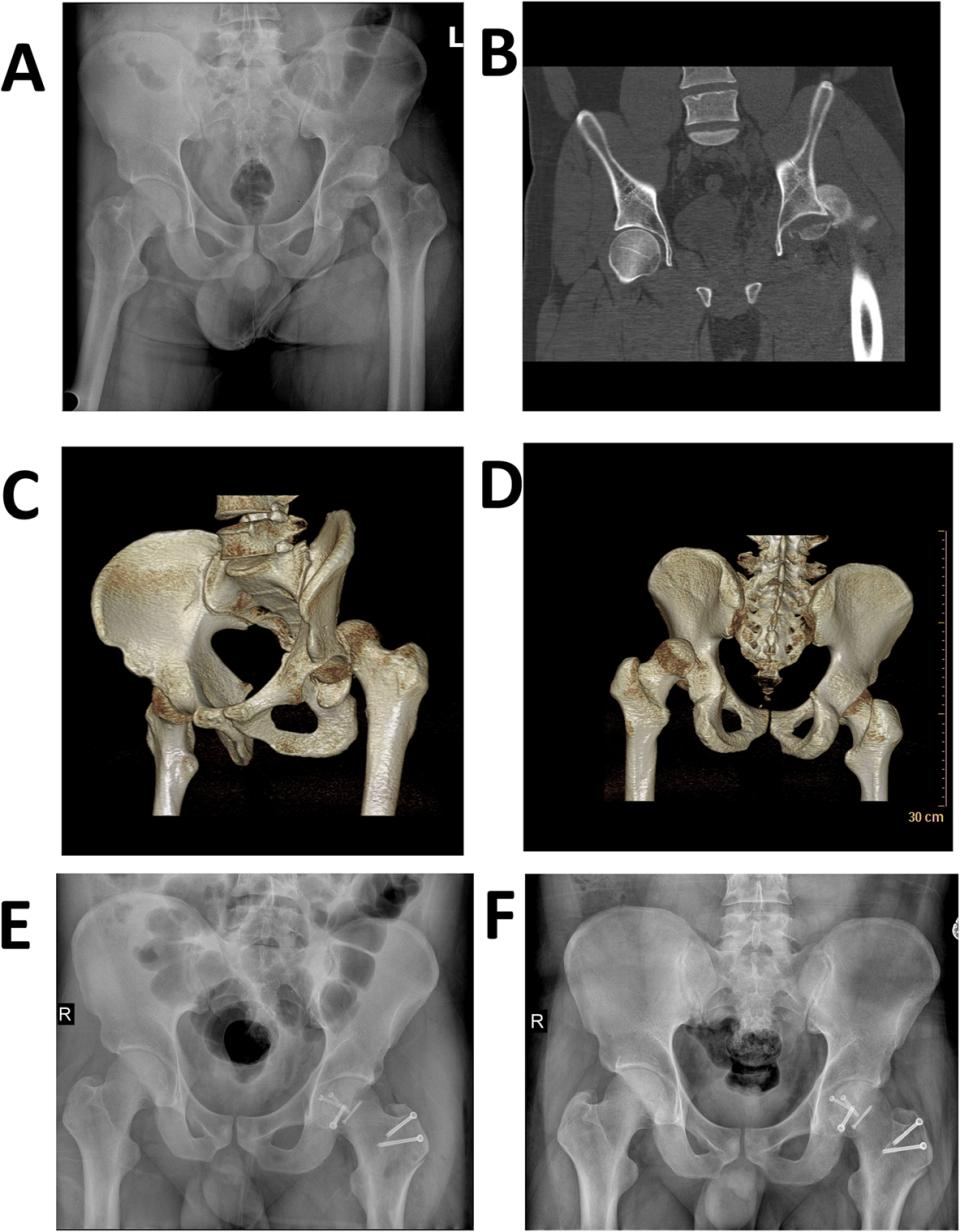
This was a 25-year-old male with femoral head fracture and hip dislocation by car accident. **a** The X-ray before operation. **b**–**d** The CT scan showed detail of the fracture. **e** The X-ray after operation. **f** the X-ray of 1 year after operation, which showed the fracture has healed and there was no AVN

For Pipkin I and II, fixation was done with 3.2-mm Herbert screws (Right, USA) or partially threaded screws under direct vision with specific attention to avoidance of weight-bearing zones and screw protrusion. The type, length, and position of the screws had been measured and determined on the 3D printing model. Associated labral tears if present were repaired with 3-mm suture anchors (Smith & Nephew, USA). For treating a Pipkin III fracture (femoral neck and femoral head fracture combined), the neck fracture should be treated first. The reposition of the femoral neck component could be performed through the same approach. In cases of Type IV fractures, we relocated the femoral head, debrided muscle tissue, and exposed the posterior wall and column. After the acetabular fractures were finished, the femoral head fractures would be handled over as above. The repaired femoral head was then gently reduced after careful retraction of the cut edges of the capsule. When performed movement in all directions of the hip is without impact and without signs of dislocation, a stable hip was obtained. Then, the capsular tear was repaired, piriformis tendon was reattached, and all muscle tears were repaired. In addition, the trochanteric osteotomy was reduced and fixation was achieved with three lag screws. The typical cases were shown in Figs. 3, 4, and 5.

### Postoperative management

The postoperative management was the same for all patients. The antibiotics were used within 3 days postoperatively. Oral indomethacin was used to prevent heterotopic ossification. Thromboembolic prophylaxis with low-molecular weight heparin was administered for 2 weeks. Patients were allowed toe touch weight-bearing for 8 weeks followed by full weight-bearing. Immediate hip range of motion exercises was initiated. Patients were recommended to perform low-impact training such as swimming or cycling when actively exercising for strengthening of the abductor muscles.

### Clinical evaluation

Functional assessment was performed using the modified Merle d’Aubigne scores [38] at last follow-up. The Merle d’Aubigne score evaluated hip function in three categories: pain, mobility and walking, each with 0–6 points. Eighteen points represented an excellent result, 15–17 points were good, 12–14 fair, and less than 12 points was regarded as a poor outcome.

### Radiological evaluation

Radiographs were taken immediately postoperative and at 8 and 12 weeks as well as 6, 12, and 24 months after surgery. The reduction of the fracture was evaluated according to Matta’s criteria [39] by measuring the residual postoperative displacements on the two plain radiographs (AP and lateral views). The highest of the three values was used to grade the reduction according to the categories: anatomical (0–1 mm of displacement), imperfect (2–3 mm), or poor (more than 3 mm).

### Complications evaluation

The incidence of complications, such as heterotopic ossification (HO) and avascular necrosis (AVN), and the need for additional surgery were also documented. The scale of Brooker et al. [40] was used to classify heterotopic ossification when present. Heterotopic ossification was classified as a complication if it interfered with joint motion.

## Results

Twelve of 17 patients (four females and eight males) were available for 2 years follow-up. As shown in Table 1, the mean follow-up was 35 months (25–48 months). Average age for the 12 patients was 39.9 ± 12.2 years. According to the Pipkin classification, the group had four patients with type I fracture, three patients with type II fracture, and five patients with type IV fracture. Eight patients sustained their injury during car accidents, two patients by falling from a high place, and the other two patients during sports. All patients underwent preoperative CT scans, 3D printed models, and simulated surgery. The 3D printed models were used as a reference for anatomical reduction of the fracture during operations. The mean operative time was 124.2 ± 22.1 min and the estimated blood loss was 437.5 ± 113.1 ml.

**Table 1:** General data

Case	Age	Gender	Pipkin type	Injury mechanism	Surgery time (min)	Intraoperative blood loss (ml)
1	26	Male	IV	Car accident	140	550
2	45	Male	II	Car accident	110	300
3	30	Female	I	Sports accident	100	400
4	48	Male	IV	Car accident	155	600
5	56	Male	II	Falling accident	130	350
6	29	Female	I	Car accident	105	400
7	44	Male	IV	Car accident	140	550
8	24	Female	II	Car accident	100	500
9	39	Male	I	Sports accident	100	350
10	54	Male	IV	Falling accident	155	550
11	28	Male	I	Car accident	110	250
12	56	Female	IV	Car accident	145	450

As shown in Table 2, functional assessment was performed at last follow-up. Most patients had good outcomes and satisfactory hip function. According to Merle d’ Aubigne scores, excellent results were achieved in six of the 12 patients; four good and two poor results occurred in the rest of the patients. On the radiograph evaluation, fracture reduction was defined as anatomical in eight patients, and imperfect in four.

**Table 2:** Follow-up data

Case	Follow-up (month)	Merle d’Aubigne score	Matta’s score	Complications
1	25	Excellent	Anatomical	Superficial wound infection
2	36	Good	Anatomical	–
3	48	Poor	Imperfect	Type II HO
4	24	Excellent	Anatomical	–
5	36	Good	Imperfect	Type II HO
6	30	Excellent	Anatomical	–
7	35	Good	Anatomical	Trochanteric osteotomy non-union
8	26	Excellent	Anatomical	–
9	40	Good	Anatomical	Type I HO
10	42	Excellent	Anatomical	–
11	37	Excellent	Imperfect	–
12	37	Poor	Imperfect	Type III HO, AVN

One superficial wound infection was found that resolved with repeated dressing and systemic antibiotics. No cases of nerve injury or deep infection were detected in the operation. All trochanteric osteotomies healed uneventfully except in one patient that developed non-union and limping. This 44-year-old man was obese and started early weight-bearing despite medical advice. He was managed by revision of the fixation 10 months after initial surgery.

One patient (56 years old, female) developed symptomatic AVN of the femoral head and underwent THA at 3 years. After THA, she regained a good hip function with the ability to return to work and almost no reduction in sports activities. Besides this patient, no other patient underwent arthroplasty. Heterotopic ossification was found in four cases (type I-1, type II-2, and type III-1); in two cases, it was considered to be causing pain or reduced range of motion and therefore classified as a relevant complication. One patient suffered with AVN as already mentioned above; in the other case, additional surgery for excision of ossification was performed.

## Discussion

Fracture of the femoral head associated is an uncommon but severe injury, with posterior hip dislocation sometimes [1]. The fracture itself as well as the subsequent complications such as heterotopic ossifcation, avascular necrosis of the femoral head, and osteoarthritis may lead to a restriction in hip function and permanent disability even in young patients. The general consensus is that operative fixation is warranted. However, controversy exists about the choice of surgical approach for femoral head fractures. Anterolateral (Watson-Jones), anterior (Smith-Petersen), and posterolateral (Kocher–Langenbeck) approaches are the most used in the literature [41]. Advantages and disadvantages of the different approaches have been the subject of previous studies [16, 42]. However, the exposure of fractures and protection of femoral head blood supply have not been well improved.

The posterior approach (K-L approach) was first reported for the treatment of femoral head fractures [43]. However, it is easy to damage the deep branch of the MFCA, leading to necrosis of the femoral head. On the other way, orientation of the fracture with more anterior and medial fracture fragment made direct screw fixation difficult especially in obese patients because of soft tissue obstruction [44]. The Ganz approach provides a safe and reliable approach for hip dislocation without injury to the blood supply of the femoral head. What is more, it offers excellent exposure with access to all areas of the femoral head and acetabulum to allow internal fixation and labral repair. Mostafa et al. [45] compared the Ganz approach versus posterior approach in the treatment of Pipkin type I and II fractures and found that Ganz approach caused less blood loss, shorter operative time, and better visualization and fixation. This makes it an attractive approach for hip resurfacing current years.

The patients treated with an anterior approach (Smith-Petersen) developed more functionally significant heterotopic ossification, although the overall functional outcome was identical [17]. Another at least theoretical disadvantage of anterior access to the hip joint might be a possible deterioration of the remaining blood supply to the femoral head after posterior dislocation and associated damage to the posterior blood vessels. So they strongly advocated posterior approaches for the surgical management of femoral head fractures [12]. However, anatomical and clinical studies do not support this theory. Stannard et al. were able to show that in comparison with an anterior approach the Kocher–Langenbeck approach was associated with an even 3.2 times higher risk of avascular head necrosis [18]. Gautier et al. did the anatomy research of the medial femoral circumflex artery based on dissections of 24 cadaver hips [13]. In the study, with the posterior approach, tenomyotomy of the external rotator muscles is necessary, which interrupts the anastomosis between the inferior gluteal artery and the deep branch of the MFCA. The deep branch itself may also be vulnerable, although there have been no cases of avascular necrosis reported after a resurfacing procedure using this approach. In addition, stable reattachment of the external rotator muscles may also be difficult. What is more, using an anterior approach, the femoral head can be dislocated safely, but inspection of the acetabulum is limited, unless the tensor fascia lata and gluteus medius are extensively detached from their origins. Ganz approach allows for a 360° view of the head and may facilitate a reduction in selected head fractures [46]. At the same time, the impact on MCFA is avoided with this approach.

It is well known that the main goal of surgical treatment for femoral head fractures with Ganz approach is to make the precise osteotomy and anatomically reduce and fix the intra-articular fragments. However, the X-ray and CT images cannot provide a comprehensive understanding for the fracture, no more for the private custom. In recent years, the application of the 3D printing applied in orthopedics was more and more common, such as pelvic fracture [31], pilon fracture, fractures of distal radius [30], and so on. Using 3D printed model, the fracture can be viewed in every direction to provide an accurate description of fracture characteristics. Besides, 3D printing model is able to help orthopedist make an individual, accurate, and reasonable surgical plan for patients, which shows its unique advantages, reducing the operation time, intraoperative blood loss, and the plasticity of plate. In this study, for all operations, the mean operative time was 124.2 ± 22.1 min, the estimated blood loss was 437.5 ± 113.1 ml. Compared with current studies, Ganz et al. [20] reported 13 patients, and the mean surgical time was 121 min for Type I or II fractures and 195 min for Type IV fractures; the mean estimated blood loss was 1334 ml in the isolated femoral head fractures and 1557 ml in head and acetabulum fractures. It was showed that using 3D printed model can reduce the operation time and intraoperative blood loss with Ganz approach for femoral head fractures. However, more RCTs with a larger sample are needed to prove the advantages of 3D printing.

No consensus exists on the safe time interval between injury and reduction of traumatic dislocation of the hip. We performed an emergency closed reduction of all patients with dislocation of the hip for less than 6 h. However, there was one patient (1/12) that developed into AVN, and it may be related to the general condition of the patient and severe lower extremity injuries. Mcmurtry and Quaile [47] showed that the joint should be relocated within 6 h otherwise the risk of avascular necrosis of the femoral head with resultant early degenerative joint disease will increase. Chen et al. [48] reported good results with surgical reduction within 12 h. Epstein et al. [12] noted that reduction within 24 h gave better results than late reduction. In general, this supported the necessity for early reduction of hip dislocation but does not define the critical time after which avascular necrosis occurs. In our opinion, the sooner the hip is restored, the better the survival of the femoral head.

In addition, clinical outcomes are reported with a variety of scoring instruments. Henle et al. [37] reported on 12 patients using the Merle d’Aubigne and Postel score. At 31 months, ten patients had good outcomes and two were considered poor. Similarly, Solberg et al. [49] reported on 12 patients with Pipkin IV fractures and noted good or excellent scores in ten. In the present study, there were six patients with excellent result, four patients with good result and two patients with poor result. It was comparable to current studies with similar good outcomes. Joint replacement was recommended for the treatment of AVN for the patient with poor outcome.

Avascular necrosis of the femoral head is the most significant complication of dislocation of the hip. AVN may be caused by damage to the blood supply at the time of injury or may be iatrogenic. Whereas initial damage to vascular structures is certainly beyond the surgeon’s control, care has to be taken during reduction maneuvers and surgery to avoid further damage to the blood supply. Surgical dislocation as described here produces an anterior dislocation using low-grade controlled trauma. The time of dislocation is much shorter than the 6-h limit which is thought to be critical after traumatic dislocations [50]. All external rotator muscles are left intact and, therefore, protect the MFCA. So, in theory, the probability of AVN in the Ganz approach is much lower than the posterior approach. Mostafa et al. [45] retrospectively reviewed patients with Pipkin type I and II femoral head fractures managed surgically through posterior Kocher–Langenbeck approach and Ganz trochanteric flip approach. Avascular necrosis of the femoral head occurred in one patient (8.1%) of trochanteric flip-approached group and two (18.1%) of posterior approach group. In our study, there was one patient with the result with AVN, and the rate is 8.3% (1/12). Henle et al. [37] made a retrospective analysis of 12 patients with femoral head fractures with posterior dislocation of the hip; there were two patients with poor outcome that developed an AVN. In Ganz et al.’s study [20], one of 13 patients developed symptomatic femoral head AVN. The rate of femoral head AVN was about the same in current studies. All the patients with AVN underwent total hip arthroplasty which was recognized as the best treatment available today.

To our knowledge, there are few prospective studies on the use of Ganz surgical dislocation for femoral head fractures [51, 52]. And this prospective study of 3D printing-based Ganz approach for treatment of femoral head fractures was implemented for the first time. Lack of controls is a limitation of this study. Although Ganz approach provides excellent exposure and satisfactory result for femoral head fractures, we should remember that the most important reasons to choose between these two approaches are the type and location of fracture, concomitant injuries, and preference of the surgeon. In the future, more randomized controlled trial is needed to prove of the superiority of this approach if it should be used as routine for displaced fractures of the femoral head.

## Conclusions

The Ganz approach provides a safe and reliable approach for femoral head fractures without injury to the blood supply of the femoral head. What is more, it offers excellent exposure, which allows for a 360° view of the fracture of femoral head, with access to all areas of the femoral head and acetabulum to allow internal fixation and labral repair. Using 3D printed model, the fracture can be viewed in every direction to provide an accurate description of fracture characteristics. And 3D printing model is able to help orthopedist to make an individual, accurate, and reasonable surgical plan for patients. Our study revealed that 3D printing-based Ganz approach could obtain excellent surgical exposure and protection of the femoral head blood supply, reduce the operation time and intraoperative blood loss, make the precise osteotomy, and anatomically reduce and fix the intra-articular fragments, which provided satisfactory results of treatment in femoral head fractures. At the same time, the probability of AVN in this surgical method was much lower than others and the impact on MCFA was avoided with the 3D printing-based Ganz approach.

## Data Availability

We do not wish to share our data, because some of the patients’ data regarding individual privacy, and according to the policy of our hospital, the data could not be shared to others without permission.

## Abbreviations

ATLS™: Adult Trauma Life Support
AVN: Avascular necrosis
CT: Computed tomography
HO: Heterotopic ossification
MFCA: Medial femoral circumflex artery
ORIF: Open reduction and internal fixation
THA: Total hip arthroplasty
PACS: Picture archiving and communication systems
K-L approach: Kocher–Langenbeck approach
